# Smartphone-Assisted High-Intensity Interval Training in Inflammatory Rheumatic Disease Patients: Randomized Controlled Trial

**DOI:** 10.2196/28124

**Published:** 2021-10-21

**Authors:** Håvard Haglo, Eivind Wang, Ole Kristian Berg, Jan Hoff, Jan Helgerud

**Affiliations:** 1 Faculty of Health and Social Sciences Molde University College Molde Norway; 2 Myworkout Medical Rehabilitation Clinic Trondheim Norway; 3 Department of Medicine University of Utah Salt Lake City, UT United States; 4 Department of Physical Medicine and Rehabilitation St. Olav’s University Hospital Trondheim Norway; 5 Department of Circulation and Medical Imaging Faculty of Medicine and Health Sciences Norwegian University of Science and Technology Trondheim Norway

**Keywords:** VO2max, maximal oxygen uptake, mobile app, cardiovascular health, quality of life, endurance training

## Abstract

**Background:**

Patients with inflammatory rheumatic diseases (IRDs) experience disease-related barriers to physical training. Compared with the general population, IRD patients are reported to have reduced maximal oxygen uptake (VO_2max_) and physical activity levels. Supervised high-intensity interval training (HIIT) is documented to counteract the reduced VO_2max_ and poor cardiovascular health associated with IRDs. However, supervised HIIT is resource demanding.

**Objective:**

This study sought to investigate if self-administered 4×4-min HIIT guided by a smartphone app (Myworkout GO) could yield similar HIIT-induced effects as standard 4×4-min HIIT performed under the guidance and supervision of health care professionals. The effects studied were on VO_2max_ and health-related quality of life (HRQoL).

**Methods:**

Forty patients (33 female patients, mean age 48 years, SD 12 years; 7 male patients, mean age 52 years, SD 11 years) diagnosed with rheumatoid arthritis, spondyloarthritis, or systemic lupus erythematosus were randomized to a supervised group (SG) or an app group (AG). Both groups were instructed to perform 4×4-min intervals with a rate of perceived exertion of 16 to 17, corresponding to 85% to 95% of the maximal heart rate, twice a week for 10 weeks. Treadmill VO_2max_ and HRQoL measured using RAND-36 were assessed before and after the exercise period.

**Results:**

VO_2max_ increased (*P*<.001) in both groups after 10 weeks of HIIT, with improvements of 3.6 (SD 1.3) mL/kg/min in the SG and 3.7 (SD 1.5) mL/kg/min in the AG. This was accompanied by increases in oxygen pulse in both groups (*P*<.001), with no between-group differences apparent for either measure. Improvements in the HRQoL dimensions of bodily pain, vitality, and social functioning were observed for both groups (*P*<.001 to *P*=.04). Again, no between-group differences were detected.

**Conclusions:**

High-intensity 4×4-min interval training increased VO_2max_ and HRQoL, contributing to patients’ reduced cardiovascular disease risk, improved health and performance, and enhanced quality of life. Similar improvements were observed following HIIT when IRD patients were guided using perceived exertion by health care professionals or the training was self-administered and guided by the app Myworkout GO. Utilization of the app may help reduce the cost of HIIT as a treatment strategy in this patient population.

**Trial Registration:**

ClinicalTrials.gov NCT04649528; https://clinicaltrials.gov/ct2/show/NCT04649528

## Introduction

Patients with inflammatory rheumatic diseases (IRDs), such as rheumatoid arthritis (RA), spondyloarthritis (SpA), and systemic lupus erythematosus (SLE), are reported to have low cardiorespiratory fitness, commonly assessed as maximal oxygen uptake (VO_2max_) [1-3].

Considering the characteristic symptoms of pain, joint swelling, fatigue, and stiffness [4-6], it is unsurprising that patients with IRDs face disease-related barriers for performing health-enhancing physical training [7-9]. Hence, patients with IRDs are observed to not only have decreased VO_2max_ compared to the general population, but also be less physically active [10], or perform physical activities with lower intensity [1]. Patients with RA, SpA, and SLE may all have these consequences [1-3]; however, SLE patients are reported to have a particularly great VO_2max_ reduction [3]. The latter IRD subpopulation is also, in general, predominantly incapacitated by physical fatigue [4], whereas for patients with SpA [6] and RA [5], pain and joint stiffness may more commonly be the leading causes of disability.

VO_2max_ is acknowledged as a strong predictor of cardiovascular disease (CVD)-related mortality and all-cause mortality [11], and it is thus unsurprising that the low VO_2max_ observed in patients with IRDs (eg, RA) is associated with an unfavorable cardiovascular profile and an increased risk of CVD [12]. IRD patients also have a higher mortality than the general population, with more than 50% of premature deaths being attributed to CVD [13].

In parallel with the relatively larger impairment of VO_2max_, patients with SLE are at a higher risk of CVD-related events and increased mortality [13,14] than RA patients [10,13] and SpA patients, where the latter subpopulation has the relatively lowest risk [10,15]. Moreover, IRD patients are at greater risk of nonfatal ischemic heart disease, which more often goes unrecognized in this patient group compared to age- and sex-matched controls [13]. Another common finding is the development of accelerated atherosclerosis [16]. Ultimately, the poor physical health status observed in the IRD population is reflected in their self-perceived health-related quality of life (HRQoL) and is particularly manifested in their experience of physical function and performance [17]. Recognizing the poor physical health status of IRD patients, training interventions aiming to effectively improve VO_2max_ are sought after.

Aerobic training, tailored to improve VO_2max_, may not only enhance physical function and performance, but also reduce the risk of CVD and all-cause mortality [11]. Furthermore, it has the potential to reduce symptom burden and inflammation in IRD patients [18]. In turn, a relief in symptoms could have a synergistic effect as disease-related factors (pain, stiffness, fatigue, disability, and quality of sleep) improve and may lower the barrier to engaging in general physical activity [8]. Aerobic training can be organized in terms of volume, frequency, and intensity, and in particular, intensity has been shown to be of critical importance for VO_2max_ improvements, with high intensity being superior to moderate or low training intensity [19]. A model for organizing aerobic high-intensity interval training (HIIT) can be 4 times 4-min work bouts carried out at 85%-95% of the maximal heart rate (HR_max_), interspaced by active recovery phases of 3 min at approximately 70% HR_max_. This model has been documented to yield effective increases in VO_2max_ in young [19], old [20], and different groups of patients [21-24]. In fact, for individuals with an aerobic capacity typical of what is observed in the general population, similar improvements in the range of approximately 0.3 to 0.4 L/min have been shown for various age groups following an 8-week HIIT intervention [25]. It has also been demonstrated to be effective and a well-tolerated mode of exercise in the IRD patient population, with similar VO_2max_ improvements as observed in healthy individuals [18,26,27]. Of importance, HIIT interventions have typically been supervised and carried out in a laboratory setting.

Unsurprisingly, supervised exercise demonstrates superior improvements in VO_2max_ compared to self-administered exercise after receiving exercise recommendations [28]. Even following initial personal instructions and free access to heart rate monitors, supervised HIIT increases VO_2max_ more than self-administered HIIT [29]. However, with recent advances in mobile technology and its availability, closer follow-up and instructions for exercising individuals may be possible. This could offer cost-effective modes of enticing patient self-management in chronic conditions [30]. Indeed, smartphone apps and associated notifications increase adherence to self-administered exercise [31]. Accordingly, apps could enhance the effectiveness of self-administered home-based exercise, and may offer a viable alternative to time- and resource-demanding supervised exercise. However, to date, results after app-guided exercise rehabilitation are equivocal. While some studies have documented improved cardiorespiratory fitness [32,33], others have not [34]. The unclear effect may, at least in part, be due to the various app designs and training interventions. Nevertheless, some studies are certainly promising with regard to the fact that app-guided training may yield some of the expected increase in VO_2max_, which is typically observed following supervised training. Thus, the aim of this study was to investigate the effect of 10 weeks of self-administered HIIT guided by an app on VO_2max_ in IRD patients, and to compare this to standard supervised HIIT. Moreover, the aim was to explore if effects on VO_2max_ were reflected in the patients’ HRQoL. Specifically, we hypothesized that both modes of exercise would increase directly assessed VO_2max_ and improve HRQoL, but that supervised HIIT would improve both outcomes more than self-administered app-guided HIIT.

## Methods

### Subjects

**Figure 1:**
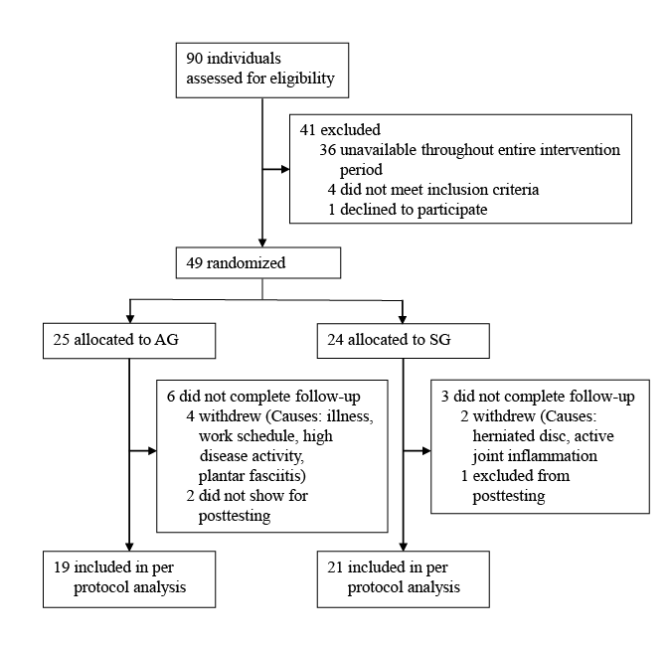
Trial flow diagram. AG: app group; SG: supervised group.

This 2-group randomized trial included 49 male and female volunteers (aged ≥18 years) diagnosed with at least one of the following IRDs: RA, SpA, and SLE. The subjects were not familiar with performing HIIT prior to inclusion in the study. They were recruited through the Norwegian Rheumatic Association and a rehabilitation clinic in Central Norway, and randomized into the following 2 groups (Figure 1): self-administered HIIT guided by an app group (AG) and standard supervised HIIT group (SG). All participants were encouraged to keep regular treatments given by the health care system, nutrition, and other physical activity habits constant throughout the study period. Sixteen of the subjects reported not engaging in any regular physical activity (AG: 7; SG: 9), while 24 of the subjects (AG: 12; SG: 12) reported being physically active 1 to 3 times per week. Prior to enrollment into the study, all participants were screened by a medical doctor for eligibility, and asked if they were able to get access to a training facility. Patients with various disease activities were included. The exclusion criteria were unstable ischemic heart disease, pregnancy, not owning a smartphone, planned surgeries influencing the training or testing, and inability to complete the testing and exercise protocol. Additionally, patients were excluded if they had other comorbid diseases, such as cerebrovascular disease, pulmonary disease, angina, diabetes type I, and hypertension, which were considered severe and/or the main limiting factor for training and testing. The participants reviewed and signed informed consent forms before participating in the study. The study was approved by the Regional Committee for Medical and Health Research Ethics in Norway and was performed in accordance with the Declaration of Helsinki.

### Study Timeline

Prior to commencement of the study, sealed opaque allocation envelopes were prepared by a third party in a 1:1 ratio. After enrollment and pretesting, participants picked and opened allocation envelopes assigning them to the AG or SG. Participants performed pretesting 1 to 3 days before the 10-week HIIT period, and posttesting was completed 2 to 5 days after the last HIIT session. Participants were instructed to not perform intensive activity 48 hours before the test days. All testing was performed by the same personnel and using the same equipment and standardized protocol before and after the training period. Testing personnel were blinded for which of the groups the subjects had been randomized to, and participants were instructed to not provide any information of their allocation. Supervising health care professionals and subjects were aware of group assignment.

### Testing of VO_2max_

Pulmonary VO_2max_ was measured using a Metamax II portable gas analyzer (Cortex Biophysik) on a treadmill calibrated for speed and inclination (Gymsport TX200). Simultaneously, heart rate was continually registered during the test using Polar RS100 (Polar Electro). In addition to determining VO_2max_ and HR_max_, performing an incremental cardiorespiratory exercise test is recommended to maximize patient safety and ensure exercise tolerance [35] before commencing a training program. Following a 6-min warm-up period at 4.0 km/h at 5% treadmill inclination, the workload was increased in increments of 1.0 km/h or 1% every minute until exhaustion. This implies that subjects with a low VO_2max_ typically performed the test walking, while subjects with a high VO_2max_ were running during the final minutes of the test. Participants received verbal encouragement and feedback from the tester throughout the test. A respiratory exchange ratio ≥1.05, in combination with a plateau in VO_2_ despite increased work rate, was used as the criterion for reaching VO_2max_ [36]. If the criterion for achieving VO_2max_ was not reached, a retest was scheduled 3 days later. If the criterion was still not met, a VO_2peak_ was reported. VO_2max_ was calculated as the mean of the 3 highest consecutive 10-second measurements. The highest heart rate measured during the last minute of the test was used as HR_max._

### HRQoL

All participants were given a generic HRQoL questionnaire (the self-administered Norwegian version of the RAND 36-Item Short-Form Health Survey [RAND-36]) at pretest and posttest. The validated Norwegian version [37] was translated from the Medical Outcome Study 36-Item Short-Form Health Survey (SF-36) [38]. RAND-36 has the same items as SF-36 with a slightly different scoring in the dimensions *bodily pain* and *general health*, and a correlation of 0.99 between the 2 scoring algorithms for these 2 items has been demonstrated [39]. The questionnaire is comprised of 8 dimensions, and scores are converted to a range from 0 to 100, with higher scores representing better health outcomes.

### HIIT

The SG and AG were instructed to undergo 2 training sessions per week on nonconsecutive days for 10 weeks. The following instructions on how to conduct HIIT sessions were given by a health care professional (SG) or a smartphone app (AG):

1. Start each session with a 6-min warm-up period at ≥5% inclination, using moderate intensity. Talking in complete sentences should still be possible (talking speed) aiming to target a rate of perceived exertion (RPE) of 13 (approximately 70% HR_max_).

2. Following warm-up, conduct the 4×4-min intervals at an intensity (speed and/or incline) that elicits heavy breathing within 2 min of each interval. It should be difficult to speak more than 2 to 3 words in a row, corresponding to an RPE of 16 to 17 (approximately 85%-95% of HR_max_).

3. The high intensity intervals should be interspaced by 3 min of active recovery with a low to moderate intensity, similar to that applied during the warm-up.

In total, each session lasted 34 min. Additionally, the following 2 rules of thumb were given: (1) at the end of each 4-min interval, being able to continue 1 more interval minute should feel possible and (2) after the fourth 4-min interval, given an active recovery period, it should feel possible to complete a fifth interval. Depending on the patients’ VO_2max_ levels, HIIT was carried out walking or running.

**Figure 2:**
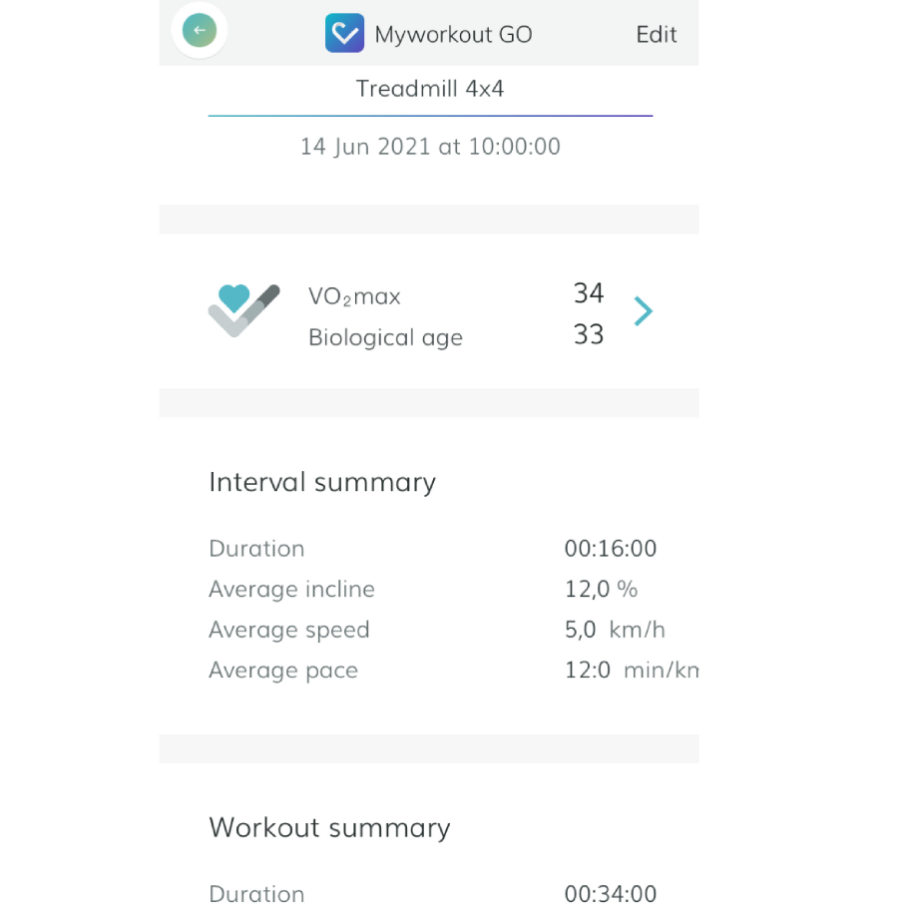
Screenshot of app (Myworkout GO) feedback following a treadmill 4×4 high-intensity interval training session for a female subject.

In order to maintain the same relative RPE intensity throughout the intervention period, absolute intensity was continuously adjusted to comply with the intensity guiding. These intensity instructions were given orally to individual subjects by a health care professional, a physiotherapist with specialization in exercise physiology, in the SG. The SG mainly trained on a treadmill in the rehabilitation clinic, which was integrated in a training facility open for the public. Some sessions (approximately one time every 2 weeks) were conducted outdoors. All intervals were carried out individually (uphill at ≥5% inclination and intensity adjusted) both indoors and outdoors to meet the targeted relative RPE. Similarly, with the intention to make the guidance type the only difference between the 2 groups, the AG conducted the training either on a treadmill indoors at a local training facility or uphill outdoors, at their own discretion, using an app (Myworkout GO, version 2.8) with written and standardized preprogrammed audio instructions, similar to the guidance given to the SG, during the HIIT. Moreover, visual display of individualized speed and incline that adapted to progression was provided by the app. After each session, the app presented performance feedback as interval work output (speed and incline), estimated VO_2max,_ and estimated biological age (Figure 2). The AG was given a scheduled posttest date at baseline, and individuals in the group were contacted once by an automated email encouraging them to comply with the planned sessions halfway in the training period, if they had logged less than 70% of the scheduled sessions. This was the only time any type of monitoring of the AG took place during the 10-week training period. With regard to safety precautions during testing and training, health care personnel had cardiopulmonary resuscitation training, and a defibrillator was available in the training facility. Although this was not available for the AG, the patients were instructed to carry out the training at daytime and contact available health care professionals and/or the medical doctor at the rehabilitation clinic by telephone if they had safety concerns. Importantly, they were instructed to contact emergency medical services if they experienced or suspected any HIIT-related adverse events. Notably, the HIIT intervention used in this study has previously been considered safe and recommendable in stable CVD patients, even in an unsupervised setting [29,40].

Considering that heart rate was not monitored during exercise sessions, intensity for both the AG and SG was estimated from work output (speed and incline) in 6 HIIT sessions and presented as a percentage of VO_2max_. Oxygen uptake (VO_2_) from the first 3 and last 3 HIIT sessions was calculated using the American College of Sports Medicine (ACSM) metabolic equations [41] and presented as a percentage of VO_2max_ from pretest and posttest. The ACSM has proposed that 60%, 80%, and 85% of VO_2max_ corresponds to 70%, 85%, and 90% of HR_max_, respectively [41].

### Statistics

The sample size calculation in this study was estimated based on the expected between-group difference in VO_2max_ at posttest. Assuming a SD of 0.2 L/kg/min with an expected mean difference of 0.2 L/kg/min between the groups, a sample of 16 subjects in each group would be required to maintain a statistical power of 0.80, with a 2-sided α of .05. However, as higher drop-out rates could be expected from patient populations, we planned to enroll 50 participants (25 in each group). Evaluation of normal distribution was performed using the Shapiro-Wilk test and Q-Q plots. All variables except HRQoL exhibited normal distribution. Per protocol analyses were performed for all the outcome measures. For most variables, paired sample *t* tests were used to detect within-group differences, and 2×2 repeated measures analysis of variance (ANOVA) with time (pre and post) and group (AG and SG) as factors was used to identify differences between groups following the training period. HRQoL was analyzed using the Wilcoxon signed-rank test to detect within-group differences, and the Mann-Whitney *U* test was used to identify between-group differences. IBM SPSS statistics software (version 26; IBM Corp) was used for statistical analyses, and GraphPad Prism software (version 8; GraphPad Software, Inc) was used to create figures. At least 70% of the planned sessions had to be completed for inclusion in the analyses. Data in the tables and text are presented as mean (SD), and data in the figures are presented as mean (SEM).

## Results

### Adherence and Characteristics

No adverse CVD events were registered during or after the VO_2max_ testing or the HIIT intervention. A total of 4 subjects withdrew from the AG; 1 due to the illness, 1 for not finding enough time to train (busy work period) in the second half of the intervention period, 1 due to a period of high disease activity, and 1 due to plantar fasciitis (week 3) possibly associated with the training. Additionally, 2 dropouts occurred from the AG as the participants forgot to appear at the posttest. Both had logged at least 16 out of the 20 planned 4×4 sessions in the app, but were unable to reschedule the posttest. Two subjects from the SG withdrew; 1 due to a low back disk herniation not related to the study and 1 due to inflammation of the knee and ankle joint possibly related to the training, as well as disease activity. Furthermore, 1 subject from the SG was excluded from the data analyses as a period of illness resulted in not complying with the planned sessions. See Figure 1 for the trial flow diagram.

**Figure 3:**
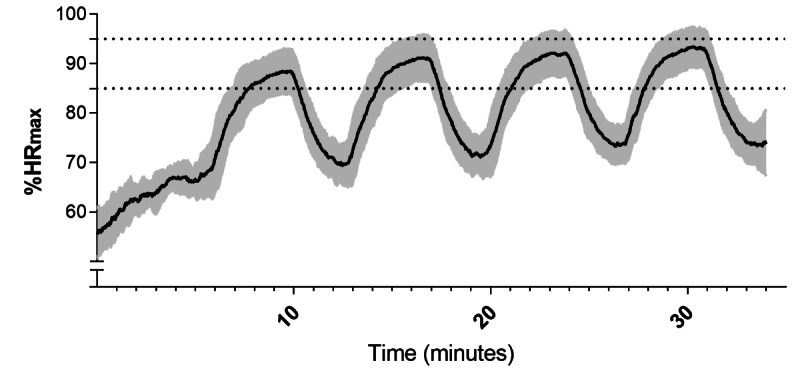
Time course of heart rate (HR) response during a 4×4 high-intensity interval training session halfway through the intervention. Subjects and the supervising physiologist were blinded to HR during the session. Intensity was guided by rate of perceived exertion. The n value is 10. The black line represents the mean. The gray error band represents SD. The area between dotted lines represents intended intensity during intervals.

Descriptive characteristics of the included participants are presented in Table 1, with no significant difference between groups observed at baseline. However, VO_2max_ (*P*=.006) and oxygen pulse (*P*=.01) were both lower in the SG than in the AG at baseline (Table 2). Both groups complied well with the planned training sessions. Among the 20 sessions, the AG performed 18.4 sessions (92% [SD 13%]) and the SG performed 19.3 sessions (97% [SD 4%]), with no apparent between-group difference (Table 1). The calculated VO_2_ from 6 HIIT sessions revealed that both the AG and SG performed these sessions with an intensity of 85%-90% of VO_2max_, with no difference between the groups. No within- or between-group difference in body mass was observed at baseline or from pretraining to posttraining. Moreover, 5 subjects from the AG did not have complete HRQoL data sets, leaving 14 completed data sets for the analyses. Halfway through the intervention period, a random sample of 10 participants in the SG performed 1 training session with heart rate monitoring (same equipment as during testing), which confirmed that the target intensities (85%-95% HR_max_) were met (Figure 3). Both the supervising physiologist and subject were blinded to heart rate data during the sampling session.

**Table 1:** Descriptive characteristics.

Characteristic			App group (n=19)		Supervised group (n=21)
Female, n (%)			14 (74)		19 (91)
Age (years), mean (SD)			48 (12)		50 (11)
Height (cm), mean (SD)			172 (9)		169 (6)
BMI (kg/m^2^), mean (SD)			26.8 (4.3)		28.3 (6.1)
**Diagnosis, n (%)**					
	Rheumatoid arthritis	4 (21)		8 (38)	
	Spondyloarthritis	11 (58)		10 (48)	
	Systemic lupus erythematosus	4 (21)		3 (14)	
Disease duration (years), mean (SD)			13 (9)		10 (9)
**Medication, n (%)**					
	Disease-modifying antirheumatic drugs	15 (79)		18 (86)	
	Nonsteroidal anti-inflammatory drugs	13 (68)		11 (52)	
Completed sessions, mean (SD)			18 (3)		19 (1)

**Table 2:** Changes in physiological parameters from pretraining to posttraining.

Parameter			App group (n=19)							Supervised group (n=21)							Between-group comparison		
			Pretraining, mean (SD)		Posttraining, mean (SD)		*P* value^a^		Pretraining, mean (SD)			Posttraining, mean (SD)		*P* value^a^		*P* value^b^			*P* value^c^
**VO_2max_^d^**																			
	Value (L/min)	2.90 (0.53)		3.15 (0.54)		<.001		2.46 (0.54)			2.74 (0.63)		<.001		.01			.46	
	Value (mL/kg/min)	36.8 (5.3)		40.5 (5.6)		<.001		31.1 (7.0)			34.7 (7.6)		<.001		.006			.97	
HR_max_^e^ (bpm)			176 (10)		177 (11)		.67		171 (16)			172 (14)		.63		.30			.88
**Oxygen pulse**																			
	Value (mL/beat)	16.6 (3.3)		17.9 (3.3)		<.001		14.3 (2.7)			15.9 (3.2)		<.001		.02			.36	
	Value (mL/kg/beat)	0.21 (0.03)		0.23 (0.03)		<.001		0.18 (0.04)			0.20 (0.04)		<.001		.01			.65	
V_E_^f^ (L/min)			81.8 (11.5)		93.6 (13.6)		<.001		73.7 (15.1)			87.8 (17.2)		<.001		.07			.29
R^g^			1.13 (0.08)		1.14 (0.07)		.47		1.15 (0.09)			1.17 (0.06)		.70		.47			.90
Body weight (kg)			79.1 (11.3)		78.2 (10.9)		.12		81.4 (19.6)			80.8 (19.2)		.10		.66			.65

^a^Within group difference from pretraining.

^b^Difference between groups at baseline.

^c^Difference between groups at posttest.

^d^VO_2max_: maximal oxygen uptake.

^e^HR_max_: maximal heart rate.

^f^V_E_: pulmonary ventilation.

^g^R: respiratory exchange ratio.

### VO_2max_ and Oxygen Pulse

**Figure 4:**
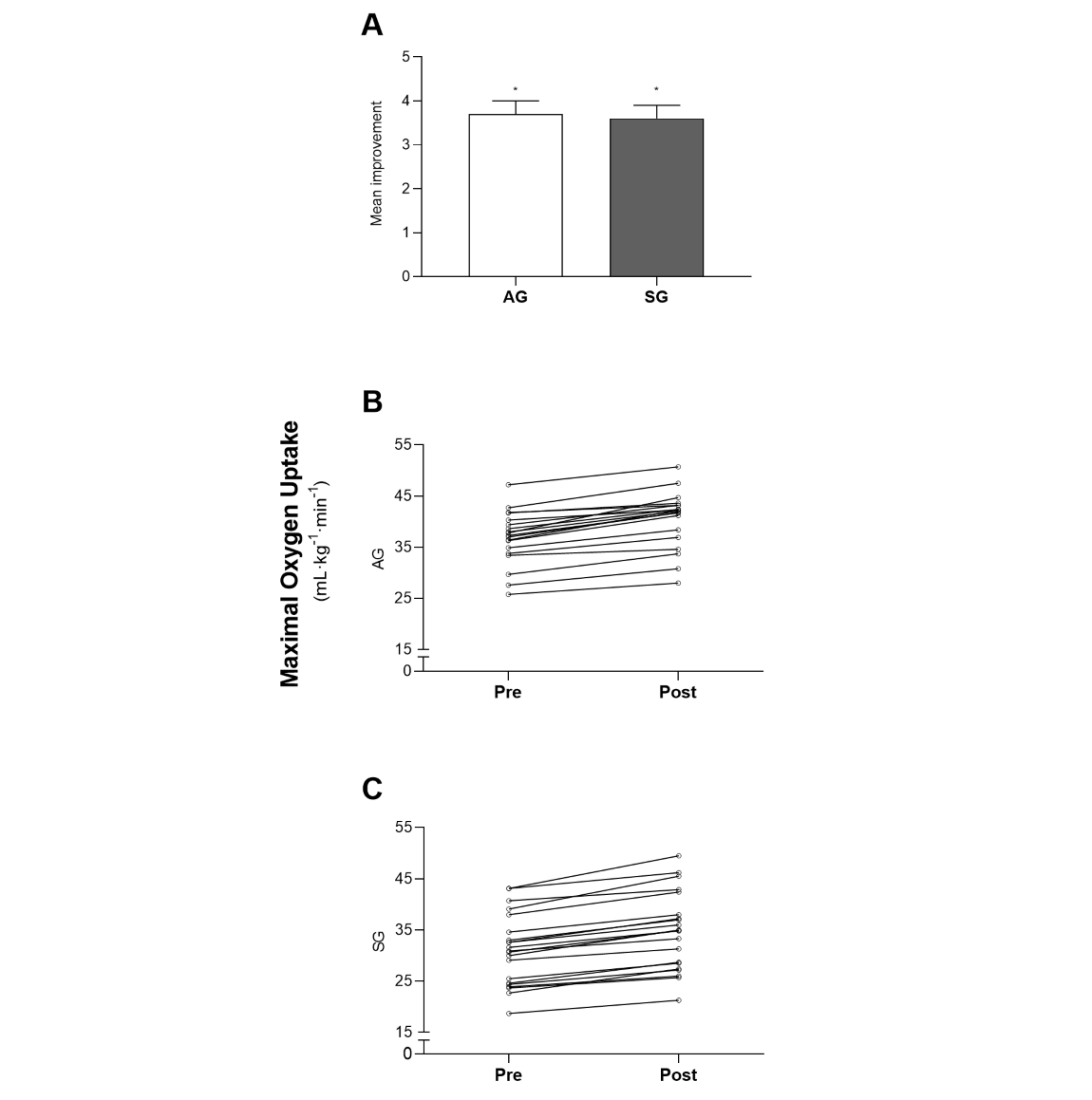
Changes in maximal oxygen uptake (VO_2max_) in mL per kg of body weight per minute after 10 weeks of high-intensity interval training. (A) Mean (SEM) change from pretraining to posttraining. (B) and (C) Individual values. AG: app group; SG: supervised group. *P<.001, within group difference from pretraining to postraining.

In accordance with the protocol, all patients reached the VO_2max_ criterion. VO_2max_ increased in both groups from pretraining to posttraining (Table 2). The AG exhibited a 10% (SD 4%) increase (*P*<.001), while the SG exhibited a 12% (SD 4%) increase (*P*<.001). No difference in improvement was apparent between the groups (Figure 4 and Table 2). After 10 weeks of HIIT, the AG and SG showed increased oxygen pulse (both *P*<.001; Table 2), and this was accompanied by increases in ventilation (both *P*<.001; Table 2).

### HRQoL

**Figure 5:**
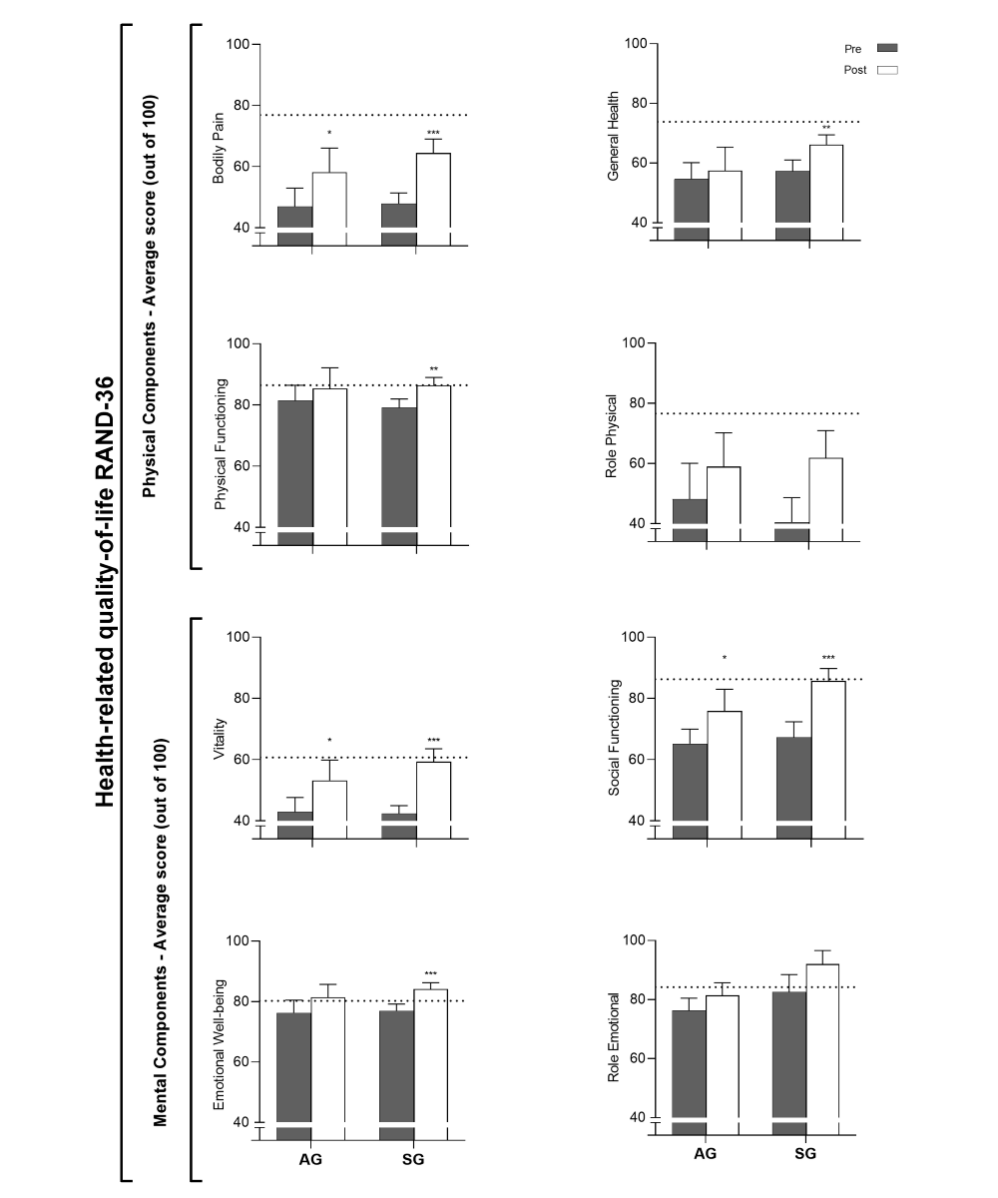
Health-related quality of life before and after high-intensity interval training. Values are presented as mean (SEM). Horizontal dotted lines represent normative data. AG: app group (n=14); SG: supervised group (n=21). *P<.05, **P<.01, ***P≤.001; significant within group difference from pretraining.

Three HRQoL dimensions improved in both groups following training. *Bodily pain* improved by 11.3 (SD 17.4; *P*=.04) in the AG and 16.7 (SD 12.6; *P*<.001) in the SG, *vitality* improved by 10.4 (SD 13.1; *P*=.01) in the AG and 16.9 (SD 17.8; *P*=.001) in the SG, and *social functioning* improved by 10.7 (SD 18.3; *P*=.04) in the AG and 18.5 (SD 15.1; *P*<.001) in the SG (Figure 5) [42]. No differences were observed between the groups following training. Additionally, the dimensions *general health*, *physical functioning*, and *emotional well-being* increased by 8.8 (SD 10.5; *P*=.003), 7.4 (SD 9.7; *P*=.004), and 7.2 (SD 6.9; *P*=.001), respectively, in the SG (Figure 5). Again, no differences were observed in these dimensions between the groups following training.

## Discussion

### Principal Findings

HIIT has been documented to effectively increase VO_2max_ and reduce the risk of CVD. However, supervised exercise treatment is time and resource demanding. With recent advances in easily available mobile technology, this may provide an opportunity to design effective, low-cost, self-management training programs for patients with chronic conditions like IRD, aiming to improve their VO_2max_. Thus, in this study, we sought to investigate if an app-guided HIIT intervention could yield some of the increase in VO_2max_ that is typically expected following supervised training. The main findings were as follows: (1) 10 weeks of HIIT (4 times with 4-min intervals) improved VO_2max_ in IRD patients; (2) the VO_2max_ increase was similar after guidance by an app and individual supervision in the rehabilitation clinic; (3) the VO_2max_ increase was accompanied by an improvement in HRQoL, and the improvement was, again, similar in the 2 groups. In contrast to our initial hypothesis, HIIT guided by an app was as effective in improving VO_2max_ and HRQoL as supervised HIIT. Short-term digital rehabilitation appears to be an excellent cost-effective alternative to supervised clinic rehabilitation of IRD patients, implying reduced risk of CVD, improved performance, and enhanced quality of life.

### VO_2max_ and the Magnitude of Improvement

Both the AG and SG showed increased VO_2max_ following 10 weeks of HIIT. However, somewhat surprisingly, the improvement in the AG (3.7 mL/kg/min) was of a similar magnitude as that observed in the SG (3.6 mL/kg/min). Importantly, both groups in this study exhibited a VO_2max_ increase comparable to what has been previously reported following supervised HIIT interventions with similar training intensity and volume in patients with active axial SpA (3.4 mL/kg/min) [43], psoriatic arthritis (3.7 mL/kg/min) [27], and RA and juvenile idiopathic arthritis (4.4 mL/kg/min) [26]. Similar HIIT-induced improvements (mean 3.8, SD 1.1 mL/kg/min) in VO_2max_ have also been reported in healthy men and women with an age comparable to that of our subjects [25].

The similar VO_2max_ improvements across the current app-based investigation and previous conventional, supervised, HIIT studies [25-27,43] strengthens the assumption that app-guided HIIT may be capable of producing an equally potent VO_2max_ increase as that of supervised HIIT. Of importance, the same instructions were given by the app as were orally provided by the instructor in the supervised training sessions. This likely contributed to the same execution of the intervals, as observed in the work output from 6 training sessions. These sessions revealed a similar HIIT intensity of 85%-90% of VO_2max_ in the AG and SG, and consequently, a similar increase in VO_2max_. Although intensity control was carried out only for the first 3 and last 3 training sessions, it gave a good indication that the app instructions or supervised guiding were perceived well when matched with the controlled intensity at the pretest and posttest. The rationale for not obtaining heart rate data from the AG during the study period was that the intention was to keep contact with the group to a minimum. The similarity in VO_2max_ improvements between previous studies and this study also indicates that both the SG and AG reached the targeted intensity by instructions and the use of RPE. Intensity is crucial, as high intensity has been previously demonstrated to be superior compared to moderate intensity to elicit improvements in VO_2max_ in both healthy individuals [19] and patients [44]. Indeed, the blinded verification tests performed halfway through the intervention in the SG (Figure 3) confirmed that the patients were trained with the intended intensity of 85%-95% HR_max_. This suggests that a heart rate monitor may not be necessary for training intensity adjustments. Accordingly, a previous study showed that the RPE during supervised heart rate monitor–guided HIIT was 16 (SD 3) on the Borg scale [27], corresponding well with our instructions targeting an RPE of 16 to 17.

### VO_2max_, Cardiac Function, CVD, and Mortality

The improvements in VO_2max_ were accompanied by increases in oxygen pulse in this study. Although an indirect measure, it may indicate a greater stroke volume of the heart [45], as previous observations have revealed that the stroke volume plays a key role when VO_2max_ is reduced or increased [46,47]. Moreover, VO_2max_ improvements following HIIT have previously been documented to be accompanied by improvements in cardiac output and stroke volume, with the arteriovenous oxygen difference remaining unchanged and no increase in HR_max_ [20]. Consequently, it is plausible that the improved oxygen pulse in IRD patients following HIIT in this study indicates improved cardiac function, which would suggest a lower risk of CVD and reduced cardiovascular and all-cause mortality [48]. Comparable to the VO_2max_ improvement in the AG and SG in this study, an increase in the VO_2max_ of 3.5 mL/kg/min has previously been documented to represent a 13% and 15% risk reduction for all-cause and CVD mortality, respectively [49].

### VO_2max_ and HRQoL

In this study, IRD patients’ VO_2max_ improvements were accompanied by greater quality of life. The HRQoL dimensions *bodily pain*, *vitality*, and *social functioning* were all enhanced in both the AG and SG following HIIT. In particular, *bodily pain* and *vitality* appeared to be severely reduced at baseline. However, following HIIT both dimensions increased closer to normative values (Figure 5), and the observed improvements indicate a reduction in symptom burden in IRD patients [50]. Interestingly, despite the SG involving social interaction between the patient and therapist, the AG exhibited a similar enhancement of these dimensions. Previous observations have shown a positive correlation between VO_2max_ and several aspects of HRQoL [51]. Thus, the positive effects of self-administered app-guided exercise on HRQoL may be explained by the increase in VO_2max_. Conversely, a VO_2max_ decrease in IRD patients has previously been associated with an impaired quality of life [52]. Moreover, worse self-reported physical functioning has also been associated with poorer HRQoL in patients with SpA [50]. Although both groups similarly showed improved VO_2max_ and most aspects of HRQoL, only the SG showed improvement in the dimension *physical functioning*. In RA patients, a minimally clinically important difference of 7.7 points in *physical functioning* has previously been described [53]. However, others have described minimally clinically important differences of 3 to 5 points [54]. The mean improvement in *physical functioning* was 3.9 (SD 10.4) and 7.4 (SD 9.7) in the AG and SG, respectively. Thus, the improvements may be below clinical importance or of marginal clinical importance in both the AG and SG. Notably, both groups were close to the normative score for the Norwegian population at baseline. Therefore, the potential for improvement was likely limited.

### HIIT: Man or Machine

Previous studies have typically reported self-administered exercise programs to yield smaller VO_2max_ improvements compared to supervised programs [29,55]. It is thus surprising that the AG and SG exhibited a similar VO_2max_ improvement in this study. The results indicate that the HIIT intervention was likely carried out similarly in the 2 training groups. Indeed, the compliance rate was not different between the 2 groups and revealed that both groups completed more than 90% of the planned sessions. This is in contrast to most previous studies documenting compliance rates to be lower after self-administered programs. For example, in a study by Cox et al [56], the compliance rate after a home-based exercise program was approximately 63% compared with approximately 84% after supervised center-based training. Interestingly, in accordance with the notion that compliance rates may explain the lower VO_2max_ response, the attenuated VO_2max_ improvement after a self-administered cardiac rehabilitation HIIT intervention in the study by Aamot et al [29] dissipated when subjects who completed less than 70% of scheduled sessions were excluded from the analysis.

The high compliance rate for HIIT in this study may be explained by the format of self-administered training. Although the AG was not supervised, automated audio instructions were given during the exercise along with written instructions within the app for guiding the intensity. Additionally, subjects consented to give researchers access to the logged sessions from the app’s server, and the awareness that they could be monitored might have made the patients commit more to the training. Halfway through the intervention, an email was sent to the subjects who had logged less than 70% of the scheduled sessions, reminding them to keep up the exercise. Thus, the AG should not be considered nonsupervised; subjects in this group were guided by the instructions in the app and were given a push notification by email. Such remote support and virtual guidance through apps have improved compliance to exercise programs in other patient groups [31,57]. For example, patients with diabetes completed as much as 95% of the planned self-administered HIIT sessions over 6 weeks when heart rate monitoring was combined with an app and an email push notification [58]. Considering compliance rates of approximately 80% [43] and 78% [27] in previous studies with IRD patients, the app certainly appears to be a viable alternative to supervised sessions, indicating that both training program compliance and execution of each training session were good.

### Safety

Previous studies in patients with IRD have reported no severe adverse cardiovascular events following incremental exercise testing or HIIT performed at an intensity of 85% to 95% HR_max_ [18,26,27,43]. In patients with coronary artery disease (CAD), the risk of cardiac incidents has been reported to be low when performing HIIT [40]. Similarly, when CAD patients followed unsupervised HIIT, no adverse cardiovascular events were observed [29]. The authors in the latter study stated that HIIT appears to be safe in CAD patients given that an incremental cardiorespiratory exercise test establishing exercise tolerance is performed before the HIIT starts. Considering previous literature and that there were no adverse events following the supervised VO_2max_ testing in either group, the initiation of both HIIT interventions was considered relatively safe in this study.

### Clinical Implications

The increasing availability of smartphone technology has introduced digital possibilities for delivering physical rehabilitation interventions. As demonstrated in this study, it appears to yield similar effects on VO_2max_ for IRD patients as effective, conventional, supervised training carried out in a rehabilitation clinic. However, it may be necessary to combine it with interactive feedback to provide successful self-administered exercise treatment. Although carried out outside the rehabilitation clinic, the utilization of apps can offer therapists detailed information on how the training is performed and the opportunity to provide detailed adjustments; however, the latter was not done in this study. Moreover, this study demonstrates that instructions and in-app information on RPE-guided exercise intensity may be an excellent alternative to heart rate monitoring of training sessions, making the administration even simpler. Rehabilitation clinics may also reach more patients through digital rehabilitation, as travel time and physical presence may limit some patients from attending treatment. Digital rehabilitation also offers a more cost-effective approach to exercise rehabilitation and may even result in enhanced patient satisfaction [59].

Interestingly, all patients in this study were able to reach VO_2max_ at pretest, implying a good tolerance for intensive training before the initiation of HIIT. For patients with confirmed or high risk of CVD or other severe concomitant diseases, unsupervised HIIT may be used in collaboration with health care professionals. Incorporating self-administered HIIT in such a way may increase patient self-efficacy and, at the same time, free up time and resources for both the patient and the treating health care professional.

### Strengths and Limitations

This study had both strengths and limitations. Allocation of patients to the AG or SG was randomized, and testers were blinded to subject allocation, which helped prevent possible selection bias. On the other hand, motivation to volunteer for participation in a research study might result in inclusion of subjects with higher internal motivation to adhere to the treatment. Notably, the group characteristics (Table 1) and VO_2max_ (Table 2) appear to be similar to data for RA and SpA populations [1,2,18,26,27], indicating that the findings may be representative of these IRD populations. Although SLE patients had typical patient characteristics in this study [3,4,60], their VO_2max_ was relatively high. Importantly, our data revealed that all 3 IRD subpopulations exhibited similar responses to HIIT and no adverse events were documented. Furthermore, this study was designed with the scope of a per protocol analysis. Hence, from an ethical perspective, posttests were only conducted with patients who were to be included in the analysis. Though this type of analysis gives great insights into the effects when subjects adhere to treatment, information that more closely reflects the clinical setting might have been obtained with an intention-to-treat analysis. This represents a limitation in this study and needs to be investigated in future research. Another limitation in this study is the lack of direct monitoring of intensity in the AG. Postexercise RPE reporting in a diary could have been done. However, such self-recorded variables often result in missing data owing to low compliance. Heart rate monitoring during home exercise would have been a viable option. Such information could be collected directly through the app, but it requires the subject to learn about wearing a monitor and to wear the monitor for each session. This may pose an added barrier to complying with the intervention. Importantly, the findings of this study indicate that such equipment or monitoring is not necessary to induce similar effects as that of supervised HIIT. Another possible limitation in this investigation is the missing data of 5 subjects in the AG for the secondary outcome (HRQoL). The reasons for not responding are unknown. Supervising self-reported questionnaire administration and requiring subjects to answer questions they do not want to or to give reasoning for not answering some questions may raise ethical concerns. Thus, controlling for such missing data is a challenge. Providing sufficient information and instructions prior to handing out questionnaires was emphasized, and further emphasis on the importance of submitting complete forms and ensuring that subjects are satisfied with their anonymity should be considered.

### Conclusion

Similar increases in VO_2max_ were observed after HIIT in IRD patients who were individually supervised or guided by oral and written instructions in the app in combination with an email reminder. The VO_2max_ improvements likely contributed to reduced risk of CVD and were accompanied by improvements in HRQoL, again with similar results between the AG and SG. Digital rehabilitation, at least in a short-term perspective, appears to be an excellent cost-effective strategy to improve the health and performance of IRD patients, and should be considered in clinical practice in the future.

## Appendix

Multimedia Appendix 1 CONSORT-eHEALTH checklist (V 1.6.1).

## Abbreviations

ACSM: American College of Sports Medicine
AG: app group
CAD: coronary artery disease
CVD: cardiovascular disease
HIIT: high-intensity interval training
HRmax: maximal heart rate
HRQoL: health-related quality of life
IRD: inflammatory rheumatic disease
RA: rheumatoid arthritis
RPE: rate of perceived exertion
SG: supervised group
SLE: systemic lupus erythematosus
SpA: spondyloarthritis
VO_2max_: maximal oxygen uptake
